# The unsynchronized changes of CT image and nucleic acid detection in COVID-19: reports the two cases from Gansu, China

**DOI:** 10.1186/s12931-020-01363-7

**Published:** 2020-04-22

**Authors:** Jing Gao, Jun-Qiang Liu, Heng-Jun Wen, Hua Liu, Wei-Dong Hu, Xia Han, Chuan-Xing Li, Xiao-Jun Wang

**Affiliations:** 1 Department of Respiratory Medicine, Gansu Provincial Hospital, No 204 Donggang West Road, Lanzhou, 730000 China; 2 Respiratory Medicine Unit, Department of Medicine & Center for Molecular Medicine, Karolinska Institutet, Stockholm, Sweden; 3 Heart and Lung Center, Department of Pulmonary Medicine, University of Helsinki and Helsinki University Hospital, Helsinki, Finland; 4 Department of Respiratory Medicine, Huining People’s Hospital, Baiyin, China; 5 Department of Respiratory Medicine, Longxi People’s Hospital, Dingxi, China; 6 Department of Internal Medicine, Gansu Provincial Hospital, Lanzhou, China

**Keywords:** COVID-19, SARS-CoV-2, CT image, Nucleic acid detection

## Abstract

The novel coronavirus disease (COVID-19) outbreak started in December 2019 in Wuhan, China, caused by severe acute respiratory syndrome coronavirus 2 (SARS-CoV-2). The CT image is used to assess the disease progress, whereas the continued two times of negative results from SARS-CoV-2 nucleic acid detection had been considered as a criterion for ending antiviral treatment. We compared the two COVID-19 cases with similar backgrounds and CT image repeated intervals under treatment. Our report highlighted the unsynchronized expression in the changes of CT image and nucleic acid detection in COVID-19, and lasting positive nucleic acid test result in patients recovered from pneumonia. It may be contributed to recognize the disease and improve prevention.

## Introduction

The novel coronavirus disease (COVID-19) outbreak started in December 2019 in Wuhan, China, caused by severe acute respiratory syndrome coronavirus 2 (SARS-CoV-2) [1, 2]. The Huanan seafood wholesale market in Wuhan, where is highly suspected as the source place of COVID-19 [3]. It has spread rapidly in China and multiple other countries [1, 4]. Over 70,000 COVID-19 cases had been confirmed until Feb 18. 2020 [5]. The CT image is used to assess the disease progress, whereas the continued two times of negative results from SARS-CoV-2 nucleic acid detection had been considered as a criterion for ending antiviral treatment. We compared the two COVID-19 cases with similar backgrounds and CT image repeated intervals under treatment. Our report highlighted the unsynchronized expression in the changes of CT image and nucleic acid detection in COVID-19, and showed the suspected SARS-CoV-2 carrier. It may be contributed to recognize the disease and improve prevention.

## Case 1 description

**Fig. 1:**
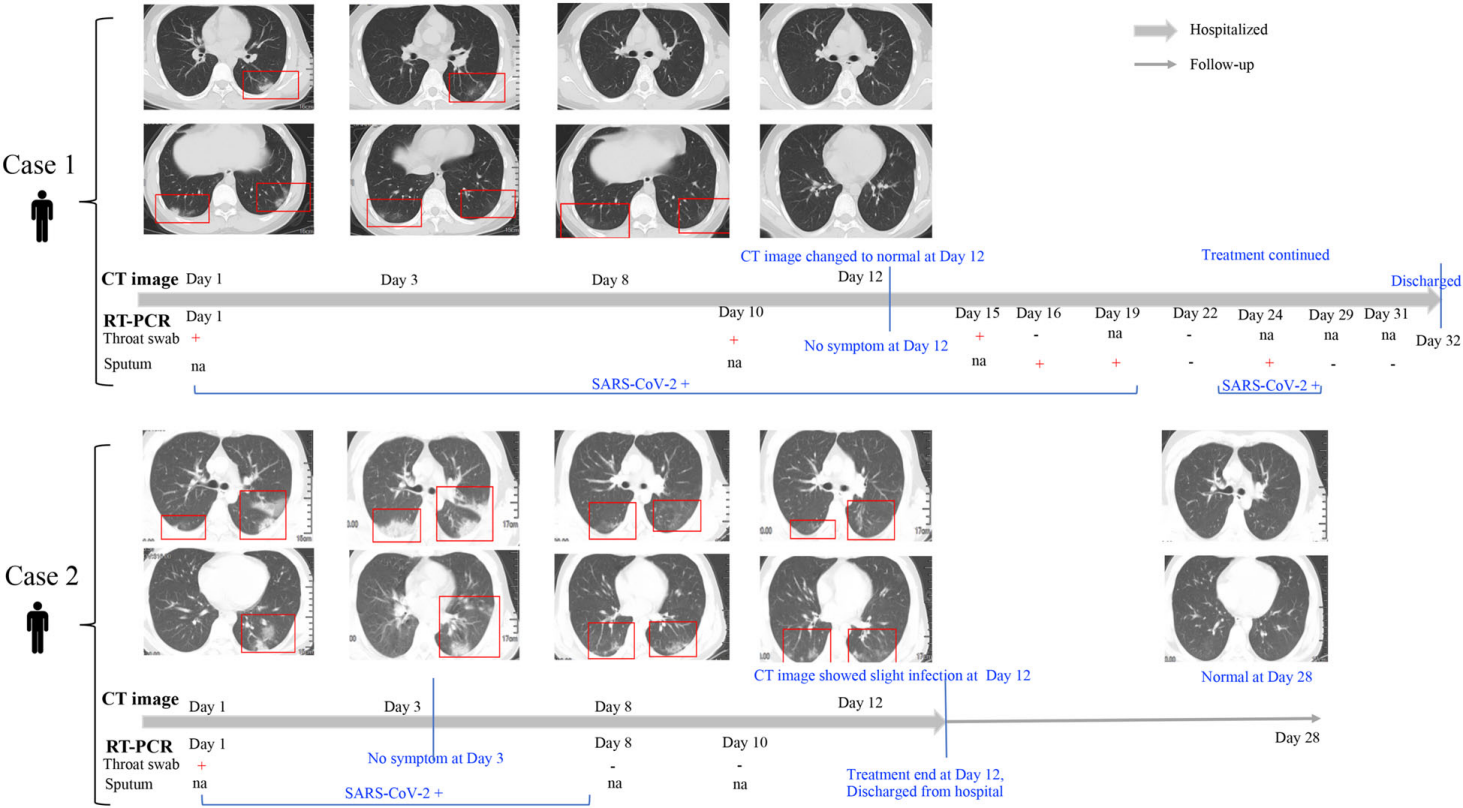
The summary of two COVID-19 cases

A 24-year-old man enrolled in his hometown hospital on Jan 22. 2020 (about 1260 km far from Wuhan), China, due to a 7-day history of mild fever, dry coughs, and weakness of unknown cause. He had been lived in Wuhan since July 2019. Fourteen days before his clinical symptom appeared, he had visited the seafood wholesale market in Wuhan. Also, he reported that he contacted with other respiratory patients with fever in Wuhan. He traveled home from Wuhan, 2 days before his presentation to the hospital. At admission (day 1), the patient reported his symptom was very mild, and his body temperature was 37.6 °C (99.7 °F), whereas coarse breath sounds of both lungs were heard at auscultation. The results of laboratory tests indicated normal white blood cell count: 5.24 × 10^9^/L. The neutrophils count declined to 46.7%, whereas C-reactive protein (CRP) was up to 11.8 mg/L. Unenhanced chest computed tomography (CT) showed multiple patchy shadows in the lower lobe of both lungs (Fig. 1). The SARS-CoV-2 nucleic acid detection of the patient’s throat swab was positive (Fig. 1). Thus, the patient was diagnosed as COVID-19. After received a day treatment with interferon inhalation (1,000,000 IU, 3/day) and azithromycin (0.5 g, 1/day), his body temperature was declined to normal 36.7 °C (98.1 °F). On day 3 from admission (day 3), the 1st repeated chest CT showed the infection range was slightly reduced in the medial segment of the right middle lobe and the dorsal segment of the left lower lobe (Fig. 1). On day 8, the 2nd repeated chest CT image showed the infection range was reduced when compared to the previous one (Fig. 1), and CRP levels was reduced to 1.3 mg/L. On day 10, the patient had less cough, and body temperature has slighter increased, but still in normal range, whereas the 2nd nucleic acid in throat swab was still positive. Thus, it’s added another antiviral medicine as lopinavir (50 mg)/ritonavir (200 mg) (2 tablets, 2 times/day) and stopped to use azithromycin from day 10 in the treatment. On day 12, the patient reported no clinical symptom, and the repeated chest CT image showed no infection in the lung (Fig. 1). On day 15, patients received the 3rd detection of nucleic acid in throat swab, it was still positive (Fig. 1). On day 16, the 4th result of nucleic acid in throat swab changed to negative, whereas it was positive in sputum (Fig. 1), thus continued the antiviral treatment with interferon inhalation and lopinavir/ritonavir. On day 19, the 5th result of nucleic acid in sputum was still positive (Fig. 1). On day 22, the nucleic acid in sputum changed to negative, whereas the result back to positive on day 24 (Fig. 1). The treatment continued. Finally, the nucleic acid detection result was changed to negative on day 29 and day 31 (Fig. 1). The patient was discharged from hospital on day 32.

## Case 2 description

A 29-year-old man enrolled in his hometown hospital on Jan 22, 2020 (about 1250 km far from Wuhan), due to a 3-day history of very mild fever and cough of unknown cause. He had been lived in Wuhan since March 2019, and contacted to the other patients with fever in Wuhan. He traveled home from Wuhan, 1 day before his presentation to the hospital. The patient reported mild symptoms at admission, since his body temperature was 37.4 °C (99.3 °F), whereas coarse breath sounds of both lungs were heard at auscultation. Laboratory tests indicated leucopenia as white blood cell count was declined to 3.38 × 10^9^/L. The white blood cell differential count showed 72.8% as neutrophils, whereas CRP and erythrocyte sedimentation rate (ESR) was elevated to 9.8 mg/L and 15 mm/h, respectively. The unenhanced chest CT image showed multiple patchy shadows in lower lobe of both lungs (Fig. 1). The SARS-CoV-2 nucleic acid in throat swab was detected as positive. Thus, the patient was diagnosed as of COVID-19 pneumonia. Since admission (day 1), the patients received the antiviral treatment with interferon inhalation (5,000,000 IU, 2 times/day) and lopinavir (50 mg)/ritonavir (200 mg) (2 tablets, 2 times/day), the antibacterial treatment with ceftazidime (2.0 g, 2 times/day), and the anti-inflammation treatment with prednisone (30 mg, 1time/day). On day 3, the body temperature declined to normal 36.3 °C (97.3 °F) and no cough. On day 8 and day 10, SARS-CoV-2 nucleic acid in throat swab were shown the negative at that two time, and the repeated chest CT image showed that the infection range was declined a lot (Fig. 1). On day 12, the infection still showed in peripheral lung, but very little (Fig. 1), thus the patient was discharged from hospital. At Feb 18, the CT image of 1st follow-up showed that no infection in his lung (Fig. 1).

## Discussion

The main finding is the unsynchronized changes in the CT image and SARS-CoV-2 nucleic acid detection in the two imported COVID-19 cases. Importantly, we highlight the need for evaluating the suspected virus carriers even after the recovery from pneumonia, who may be asymptomatic with no positive result in laboratory test. The PCR positive result in the 1st young male patient lasted nearly 20 days after his pneumonia recovery, suggested a proportion of recovered patients still may be asymptomatic virus carriers. When SARS-CoV-2 enters humans, the interplay between virus and host antiviral defence is a governed factor for outcome of infection. Asymptomatic carriers might arise when host antiviral defence is either strong or decoupled, thus asymptomatic shedding might occur when the immune response effectively limits but could not enough to completely block SARS-CoV-2 replication [6]. Thus, the continued treatment and isolated is needed, since the risk of transmitting to others still exist. On the other hand, it is the need to evaluation the effect of the long-time antiviral treatment and side effect, especially for positive SARS-CoV-2 nucleic acid patient without any pneumonia. The unsynchronized result of nucleic acid detection between throat and sputum sample were shown in the 1st patient in our study. First patient showed the virus PCR-negative throat swab on day 16, whereas the result in sputum was continued to be positive until day 24. Thus, we supposed that the different viral load shown between lower respiratory tract than upper respiratory tract. Hase. et al. study reported that a COVID-19 case diagnosed by PCR-positive lower respiratory specimen, but with PCR-negative throat swabs [7]. Although the knowledge of viral loads in COVID-19 is limited, the high viral loads of lower respiratory tract specimens had been reported in severe acute respiratory syndrome (SARS) and Middle East respiratory syndrome (MERS) [8, 9]. Our results not only confirmed the necessary of multiple times test in COVID-19, but also highly recommended to use multitype samples for the disease evaluation, especially lower airway. To collect the induced sputum may be recommended for the patients with dry cough or no symptom. We also noticed that the unsynchronized expression could also show in the diagnosis stage. In our cases, the patient had very less symptom, whereas the CT image showed a clearly infection. This unsynchronized expression may help clinician to arrange the examination plan for suspected COVID-19 patient. The choice of antiviral medicine for COVID-19 is still controversial, especially no specific anti-SARS-CoV-2 medicine is available. However, we noticed that the 2nd patient got quicker recovery compared to the 1st patient, which may relate to the use of double antiviral medicines at early stage. Olfactory disturbance or taste disorder was reported in COVID-19 patients [10], and these symptoms did not been reported by our patients at hospital or follow-up. The study was limited to a small number of patients, which could not identify the representative and generalizable in COVID-19.

## Conclusion

The unsynchronized changes in the CT image and SARS-CoV-2 nucleic acid detection in the two imported COVID-19 cases. Lasting positive nucleic acid test result shown in patients recovered from COVID-19. Our report would be contributed to aid clinicians in a more personalized management.

## Data Availability

No

## Abbreviations

COVID-19: Novel coronavirus disease
SARS-CoV-2: Severe acute respiratory syndrome coronavirus 2
RT-PCR: Real-time polymerase chain reaction
CT: Computed tomography
CRP: C-reactive protein
ESR: Erythrocyte sedimentation rate
SARS: Severe acute respiratory syndrome
MERS: Middle East respiratory syndrome
